# Risk predictors of advanced hepatic fibrosis in patients with nonalcoholic fatty liver disease – a survey in a university hospital in Brazil

**DOI:** 10.20945/2359-3997000000514

**Published:** 2022-09-20

**Authors:** Thaís Grecca Andrade, Luana Cavalcanti Dias Xavier, Fernanda Fernandes Souza, Roberta Chaves Araújo

**Affiliations:** 1 Universidade de São Paulo Faculdade de Medicina de Ribeirão Preto Ribeirão Preto SP Brasil Faculdade de Medicina de Ribeirão Preto, Universidade de São Paulo, Ribeirão Preto, SP, Brasil; 2 Universidade de São Paulo Faculdade de Medicina de Ribeirão Preto Hospital das Clínicas Ribeirão Preto SP Brasil Divisão de Gastroenterologia do Hospital das Clínicas da Faculdade de Medicina de Ribeirão Preto – Universidade de São Paulo, Ribeirão Preto, SP, Brasil

**Keywords:** Fatty liver disease, liver fibrosis, metabolic syndrome

## Abstract

**Objective:**

Describe the clinical profile of patients with biopsy-proven non-alcoholic fatty liver disease (NAFLD) and analyze the risk predictors of hepatic fibrosis in outpatient follow-up at a university hospital.

**Subjects and methods:**

Demographic, clinical and laboratory data of a cohort of 143 patients with biopsy-proven NAFLD were retrospectively analysed under univariate analyses. Diagnostic accuracy, determined by AUROC, was evaluated for variables that showed a significant difference in univariate comparison analysis and diagnostic performances were determined by sensitivity and specificity.

**Results:**

The mean age of studied patients were 48 years, 66.4% of them were women. Age, presence of diabetes mellitus, hypertension, metabolic syndrome and laboratory variables such as AST/ALT ratio, GGT, platelet count and fasting glucose were significantly associated with advanced fibrosis. FIB-4 and NAFLD fibrosis score (AUROC 0.82 and 0.89, respectively) outperformed APRI (AUROC 0.73) for advanced liver fibrosis and cirrhosis (P of 0.04).

**Conclusion:**

In our study, metabolic syndrome, diabetes, hypertension, AST/ALT ratio, GGT, platelet count and fasting glucose were associated with hepatic fibrosis in patients with NAFLD. The non-invasive tests FIB-4 and NAFLD fibrosis score showed the best accuracy to stratify disease severity.

## INTRODUCTION

Non-alcoholic fatty liver disease (NAFLD) is one of the most common etiologies of liver disease, with a worldwide prevalence of 25% (1). It is strongly associated with obesity, insulin resistance, type 2 diabetes, hypertension, dyslipidemia and metabolic syndrome (1–3). Recently, the term metabolic associated fatty liver disease was proposed in order to better describe the contribution of cardiometabolic risk factors to the development and progression of liver disease, and help in patient stratification and disease management (4,5), but is not yet the currently accepted nomenclature.

NAFLD comprises a spectrum of diseases, ranging from isolated non-inflammatory steatosis, defined by lipid accumulation in the cytoplasm of more than 5% of hepatocytes, to non-alcoholic steatohepatitis (NASH), characterized by specific histological changes, such as steatosis, inflammatory infiltrate and hepatocyte ballooning with or without fibrosis (6). At least one third of patients with NAFLD will progress to NASH, a more advanced form of disease, with potential progression of liver fibrosis, which may complicate with cirrhosis, hepatocellular carcinoma and need for liver transplantation (2). These patients have an increased overall mortality and mortality associated with liver disease compared to the general population. This risk rises exponentially as the fibrosis stage progresses from stage 0 to stage 4 (7). There are some known risk factors for disease progression such as type 2 diabetes mellitus (T2DM), which is associated with a more than two-times increased risk of advanced fibrosis, cirrhosis-related complications, and liver disease mortality. Obesity, lipid abnormalities, and hypertension are also associated with an increased risk of severe liver disease, although the effect sizes are smaller than for T2DM (4).

Epidemiological, clinical, genetic and environmental factors contribute to the heterogeneity of NAFLD presentation. The purpose of this study is to describe the clinical profile of patients with biopsy-proven NAFLD and to evaluate the risk predictors of advanced hepatic fibrosis and cirrhosis in this population. As it is a highly prevalent disease, with different local realities, studies as this one serve as a basis for knowledge of the local reality and provide important information for the development of public health programs.

## SUBJECTS AND METHODS

This is a retrospective cross-sectional study on patients with biopsy-proven NAFLD, in the period of 2000 to 2018, followed-up at the Liver Metabolic Disease Outpatient Clinic of the University Hospital of the Ribeirão Preto Medical School, University of São Paulo, Brazil. The local ethics committee approved the study (Certificate of Presentation for Ethical Appreciation #12396519.5.0000.5440).

Patients aged > 18 with biopsy-proven NAFLD, as defined by Brunt and cols., were included (8). An experienced pathologist in the hepatology field analyzed the biopsies, samples described as non-representative were not considered. Exclusion criteria were a record of alcohol abuse with a threshold > 20 g/day in women and > 30 g/day in men, human immunodeficiency virus infection and other liver diseases such as hepatitis B, hepatitis C, autoimmune hepatitis, hemochromatosis, Wilson disease, primary sclerosing cholangitis, primary biliary cholangitis, alpha-1 antitrypsin deficiency and drug-induced liver injury.

Clinical and laboratorial data were collected within a maximum period of six months before or after biopsy, and they are as follows: age, race, gender, weight, height, body mass index (BMI), and presence of smoking, alcoholism, hypothyroidism, T2DM, prediabetes, insulin resistance, systemic arterial hypertension, dyslipidemia and metabolic syndrome. BMI was classified according to the World Health Organization and metabolic syndrome was diagnosed following the International Diabetes Federation criteria (9). Alanine aminotransferase (ALT), aspartate aminotransferase (AST), alkaline phosphatise (ALP), gamma glutamyl transpeptidase (GGT), total cholesterol, HDL, LDL, triglycerides levels (TG), serum albumin, ferritin, platelet count, fasting plasma glucose, glycated haemoglobin (HbA1c), HOMA-IR and thyroid hormone (TSH) were also assessed. ALT normal values were less than 19 U/L and 30 U/L for women and men, respectively (10).

The dependent variable was liver fibrosis and to evaluate the association of histological characteristic with clinical-demographic and laboratory variables, absent or mild/moderate fibrosis (grades 0+1+2) versus advanced liver fibrosis/cirrhosis (grades 3+4) were analyzed.

Non-invasive tests based on clinical and biochemical variables developed to stage liver fibrosis were calculated for patients who had all variables in medical records. We calculated three scores: The Fibrosis-4 (FIB-4) index, NAFLD fibrosis score (NFS) and aspartate aminotransferase-to-platelet ratio index (APRI), using the original formulas (11–13). FIB-4: age (years) × AST (U/L) / platelets (10^9^/L) x √ALT (U/L). NFS: -1.675 + 0.037 × age (years) + 0.094 × BMI (kg/m^2^) + 1.13 × diabetes (yes = 1, no = 0) + 0.99 × AST/ALT ratio - 0.013 × platelet count (x10^9^/L) - 0.66 × albumin (g/dL). APRI index: [(AST/upper limit of the normal AST range) x 100]/Platelet count.

Categorical variables were presented as frequency and percentage, and continuous variables as mean and median values, standard deviation, and range. A chi-square analysis and a chi-square for linear trend were used to compare categorical variables, and the continuous variables were analyzed using the non-parametric Kruskal-Wallis with post hoc test of Dunn and Mann-Whitney tests. Diagnostic accuracy was evaluated by determining the area under the receiver-operator characteristics curve (AUROC) for variables that showed a significant difference in univariate group comparison analysis, after calculating the cut-off with highest Youden Index (14). Diagnostic performances were determined by sensitivity (Se), specificity (Sp), positive predictive value (PPV) and negative predictive value (NPV). Pairwise comparisons of AUROC’s were performed for fibrosis models using the DeLong method (15). For this comparison, only patients who had all data for calculating the non-invasive tests were included. A *P* value less than 0.05 was considered significant. Statistical procedure interpretation of data was performed using Statistical Package for Social Sciences (SPSS) version 23.0 for Windows.

## RESULTS

### Characterization of the sample

A total of 219 patients with biopsy-proven NAFLD were reviewed, 76 were excluded due to the following reasons: alcohol consumption more than the considered limit (19.7%), viral hepatitis (50%), presence of another hepatopathy (21.1%) and lack of data in medical records (9.2%). Finally, 143 patients were included, 95 were female, aged 19-68 years (mean = 48 ± SD 11.8 years).

Table 1 shows laboratory data and frequency of known risk factors for NAFLD in the study population. There were 91 patients with obesity, 53 (40.2%) of them had grade I obesity, 27 (20.5%) grade II, and 11 (8.3%) grade III. The waist circumference was analyzed in 52 of 143 patients and ranged from 86 to 145.5 cm, found compatible with metabolic syndrome in 51 cases (98.1%). The majority of patients, 71 (74.7%), had high cardiovascular risk, stratification based on the Brazilian Guideline on Dyslipidemias and Atherosclerosis Prevention (16). Most patients were not smokers, only 18 (12.9%) claimed to have this habit at the time of biopsy.

**Table 1:** Laboratory data and comorbid conditions associated with nonalcoholic fatty liver disease present in patients evaluated

Variables	N	% (Median – IQR)
AST (U/L)	128	37.6 – 28.8
ALT (U/L)	129	55 – 52.3
AST/ALT	127	0.7 – 0.3
GGT (U/L)	99	65 – 81
ALP (U/L)	118	187 – 78.5
Cholesterol (mg/dL)	116	197 – 63.7
HDL (mg/dL)	112	41 – 12
LDL (mg/dL)	106	117 – 49.7
Triglycerides (mg/dL)	111	169 – 129.9
Albumin (g/dL)	78	4.4 – 0.3
Ferritin (ng/mL)	93	232 – 290
Platelet count (x 10^9^/L)	118	226 – 90.2
Glucose (mg/dL)	114	97 – 32.9
HbA1c	42	7.2 – 3
TSH	42	2.1 – 1.9
Diabetes mellitus	61/137	44.5
Hypertension	71/138	51.4
Dyslipidemia	101/136	74.3
Obesity	91/132	68.9
Increased abdominal waist	51/52	98.1

*N:* number of individuals; %: percentage; IQR: interquartile range; AST: aspartate aminotransferase; ALT: alanine aminotransferase; GGT: gamma-glutamyl transpeptidase; ALP: alkaline phosphatise; HDL: high-density lipoprotein; LDL: low-density lipoprotein; HbA1c: glycated haemoglobin; TSH: thyroid hormone.

### Non-invasive fibrosis assessment and liver biopsy

Non-invasive scoring systems were evaluated in patients who had all variables in their medical record. FIB-4 index was calculated in 112 patients, 75 (67%) had a score < 1.3 and 5 (4.5%) ≥ 3.25. APRI’s determination included 118 individuals, 61 (51.7%) had a score < 0.5 and 9 (7.6%) > 1.5. NFS was estimated in 67 patients: 27 (40.3%) had a score < -1.475 and 7 (10.4%) > 0.675.

We classified liver biopsies according to the severity of steatosis and fibrosis (8). Liver steatosis presented the following results: grade 1 in 12 (17.6%) patients, grade 2 in 31 (45.6%), grade 3 in 25 (36.8%), and in 75 patients (52.4%) steatosis was not graded. One hundred forty-one patients (98.6%) had histologic criterias for NASH. Fibrosis classification showed the following results: absence of fibrosis (F0 = 1.4% of the patients), zone 3 perisinusoidal fibrosis (F1 = 66%), as above with portal fibrosis (F2 = 15.6%), as above with bridging fibrosis (F3 = 10.6%) and cirrhosis (F4 = 6.4%).

### Association between clinical-demographic and laboratory data and the severity of liver disease

Age, T2DM, hypertension, the presence of metabolic syndrome and laboratory data, such as AST/ALT ratio, GGT, platelet count and fasting blood glucose were significantly associated with advanced liver fibrosis and cirrhosis as well as the non-invasive scoring systems, as shown in Tables 2 and 3, respectively.

**Table 2:** Association of clinical-demographic and laboratory data with the degree of hepatic fibrosis

			Fibrosis degree*		*P*
			F0 + F1 + F2	F3 + F4	
Age		Frequency (*n*)	117	24	0.001
		Median	49	55	
		IQR	18.5	9.5	
Diabetes mellitus					
	Present	Frequency (*n*)	47	14	0.03
		Valid percentage (%)	41.2	66.7	
	Absent	Frequency (*n*)	67	07	
		Valid percentage (%)	58.8	33.3	
Hypertension					
	Present	Frequency (*n*)	53	17	0.003
		Valid percentage (%)	46.1	81	
	Absent	Frequency (*n*)	62	04	
		Valid percentage (%)	53.9	19	
Metabolic syndrome					
	Present	Frequency (*n*)	71	18	
		Valid percentage (%)	67	90	
	Absent	Frequency (*n*)	35	02	
		Valid percentage (%)	33	10	
AST/ALT ratio		Frequency (*n*)	105	20	0.008
		Median	0.7	0.9	
		IQR	0.2	0.4	
GGT		Frequency (*n*)	99	19	0.007
		Median	56	97	
		IQR	67.6	126	
Platelets count (x 10^9^/L)		Frequency (*n*)	99	18	0.004
		Median	236	194.5	
		IQR	85	87.7	
Fasting blood glucose		Frequency (*n*)	95	17	0.03
		Median	97	137	
		IQR	28	94.1	

*n:* number of individuals; IQR: interquartile range;

* Fibrosis degree: F0: absence of fibrosis; F1: zone 3 perisinusoidal fibrosis; F2: as above with portal fibrosis; F3: as above with bridging fibrosis; F4: cirrhosis; *P* value; AST/ALT ratio: aspartate aminotransferase/alanine aminotransferase ratio; GGT: gamma-glutamyl transpeptidase.

**Table 3:** Association between non-invasive scores with the degree of hepatic fibrosis

	Fibrosis degree*	Frequency (*n*)	Median	IQR	*P*
FIB-4	F0 + F1 + F2	94	1.02	0.77	<0.001
	F3 + F4	17	2.28	1.59	
APRI	F0 + F1 + F2	98	0.46	0.42	0.003
	F3 + F4	18	0.76	0.72	
NFS	F0 + F1 + F2	54	-1.50	1.77	<0.001
	F3 + F4	12	0.37	1.23	

*n*: number of individuals; IQR: interquartile range; *P* value; FIB-4: Fibrosis-4 index; APRI: AST/platelet ratio index; NFS: NAFLD fibrosis score.

* Fibrosis degree: F0, absence of fibrosis; F1, zone 3 perisinusoidal fibrosis; F2, as above with portal fibrosis; F3, as above with bridging fibrosis; F4, cirrhosis.

AUROC and detailed test performances are shown in Table 4. The diagnostic performance of non-invasive scores for its cut-offs recommended in the literature is detailed in Table 5. For excluding advanced liver fibrosis the results were as follows: at a cut-off of -1.455, NFS had a Se of 1 (0.74-1), Sp of 0.50 (0.36-0.64), PPV of 0.31 (0.17-0.48) and NPV of 1 (0.87-1). At a cut-off of 1.3, FIB-4 index had a Se of 0.76 (0.50-0.93), Sp of 0.65 (0.54-0.74), PPV of 0.28 (0.16-0.43) and NPV of 0.94 (0.85-0.98). Regarding APRI, at a cut-off of 0.5, its Se was 0.83 (0.59-0.86), Sp 0.56 (0.46-0.66), PPV 0.26 (0.15-0.39) and NPV 0.95 (0.86-0.99).

**Table 4:** Area under the receiver-operator characteristics curve, cut-point values and diagnostic performance of demographic, laboratory variables and scores for advanced liver fibrosis and cirrhosis

	AUROC	Cut-off value	Sensitivity (%)	Specificity (%)	95% Confidence Interval	P*
Age	0.67	51	75	55	56.7-77.4	0.009
AST/ALT ratio	0.70	0.8	60	79	57.4-83.1	0.004
GGT	0.69	60	84	52	57.1-82.2	0.007
Platelets count (x 10^9^/L)	0.71	205	61.1	73.7	58.4-84.2	0.004
Fasting blood glucose	0.67	115	64.7	74.7	50.7-83.3	0.03
FIB-4	0.82	1.69	70.6	87.2	71-93.7	<0.001
APRI	0.71	0.52	83.3	60.2	58-85.5	0.003
NFS	0.90	-0.42	91.7	79.6	82-97.9	<0.001

AUROC: area under the receiver-operator characteristics curve; *P* value: in this case, it demonstrates that the variable provides better prediction than the null hypothesis; AST/ALT ratio: aspartate aminotransferase/alanine aminotransferase ratio; GGT: gamma-glutamyl transpeptidase; FIB-4: Fibrosis-4 index; APRI: AST/platelet ratio index; NFS: NAFLD fibrosis score.

**Table 5:** Diagnostic performance of fibrosis scores according to its cut-offs recommended in the literature in patients who had all the variables in medical records for its calculation

	Cut-off value	Sensitivity % (95% IC)	Specificity % (95% IC)	PPV % (95% IC)	NPV % (95% IC)
FIB-4	1.3	76 (50-93)	65 (54-74)	28 (16-43)	94 (85-98)
	2.67	19 (10-56)	96 (89-99)	46 (27-67)	94 (87-98)
	3.25	18 (4-438)	97 (91-99)	50 (12-88)	87 (79-963)
APRI	0.5	83 (59-86)	56 (46-66)	26 (15-39)	95 (86-99)
	1.5	22 (6-48)	9 (87-98)	40 (12-74)	87 (79-93)
NFS	-1.455	100 (74-100)	50 (36-64)	31 (17-48)	100 (87-100)
	0.675	42 (15-72)	94 (85-99)	63 (24-91)	88 (75-95)

95% IC: 95% confidence interval; %: percentage; PPV: positive predictive value; NPV: negative predictive value; FIB-4: FIB-4 index; APRI: AST/platelet ratio index; NFS: NAFLD fibrosis score.

FIB-4 and NFS (AUROC 0.82 and 0.89, respectively) outperformed APRI (AUROC 0.73) for advanced liver fibrosis and cirrhosis with a *P* of 0.04. There was no significant difference between FIB-4 and NFS AUROC’s.

## DISCUSSION

NAFLD is one of the most common chronic liver disorders, with a global prevalence of around 25% (1,4). It has a wide spectrum of clinicopathological severity, which is influenced by multiple factors including age, gender, hormonal status, ethnicity, diet, alcohol intake, smoking, genetic predisposition, the microbiota and metabolic status (5). NAFLD can be diagnosed in any age group, including children, however, there is a higher prevalence between 40 and 49 years (17). We found a predominance of the disease in middle-aged women and an association between older patients and higher degree of liver fibrosis (*P* = 0.001). The correlation with gender is controversial: early studies claimed that NAFLD was more common in women, but the latest concluded the opposite (18). Usually, prevalence is lower in women predominantly at earlier disease stages, whereas disease frequency increases in postmenopausal women (19). The increased frequency in postmenopausal women and the longer duration of estrogen deficiency are associated with a greater chance of fibrosis in this group (20). Although not completely understood, the behavior of the disease is probably associated with sexual differences in metabolic risk factors, adiposity and body fat distribution, and women tend to predominate in central obesity in the postmenopause (21). Many factors are implicated in the relationship between ageing and liver fibrosis, such as decline in hepatic blood flow, hepatic volume, and liver function, which occur with age. Besides that, changes in body composition, including a decrease in muscle mass, an increase in abdominal adiposity and ectopic fat deposition, with increases in insulin resistance and prevalence of the metabolic syndrome can also be associated (5).

NAFLD is strongly associated with obesity, dyslipidemia, T2DM, and metabolic syndrome. On average, 45% and 70% of the studied patients had diabetes and metabolic syndrome, respectively, and these conditions were associated with advanced liver fibrosis and cirrhosis, as well as fasting blood glucose. T2DM in NAFLD is a risk factor for progression to NASH, and is associated with a more than two-times increased risk of advanced fibrosis, cirrhosis-related complications, and liver disease mortality (4,22). Atherogenic dyslipidemia characterized by low HDL values and elevated triglycerides, is the most common form of dyslipidemia in patients with NAFLD (23). In this study, 71% of the evaluated patients had low HDL and hypertriglyceridemia was present in 58% of the patients who had this data recorded. The strong link between insulin resistance and lipid metabolism is well known. Insulin resistance facilitates an increase in the flow of free fatty acids, which raises the production of very low-density triglycerides and lipoproteins and trigger lipid oxidative stress, all closely associated with the development of steatohepatitis (24).

The prevalence of hypertension is significantly higher in individuals with NAFLD compared to the general population. About 51% of the individuals evaluated were hypertensive. NAFLD can induce systemic effects such as inflammation, activation of the renin-angiotensin system, activation of the sympathetic system and insulin resistance, which are pathophysiological mechanisms for the development of hypertension (25,26). In the present study, there was a large number of individuals with obesity (63.3%) and almost all patients who had their waist circumference assessed had values above normal (98.1%). Obesity is clearly associated with fatty liver, although not all patients with obesity develop NAFLD and many individuals with this diagnosis have normal body weight (27). Current consensus suggests that the main determinant of NAFLD risk would not be the amount of fat, but probably its distribution, since greater amounts of visceral fat in relation to peripheral and subcutaneous adipose tissue are associated with higher metabolic risk and, consequently, directly linked to liver inflammation and fibrosis. This mechanism occurs by increasing the flow of fatty acids to the liver through the portal vein. Increased waist circumference is considered a marker of increased visceral adiposity (5,28).

About 85% of our patients had aminotransferases levels above normal, and AST/ALT ratio was associated with higher degree of fibrosis (*P* = 0.008). Mild to moderate elevations of serum aminotransferases are the most common laboratory changes found in patients with NAFLD (29). Elevations are usually 1 to 4 times the upper limit of normality, with ALT levels higher than AST in mild fibrosis, and the opposite may occur in advanced stages of the disease (17). In our study GGT was associated with advanced liver fibrosis and cirrhosis (*P* = 0.007). This enzyme has a prooxidant activity and a modulating influence on endothelial dysfunction. It is associated with metabolic syndrome and is often elevated in patients with NAFLD. There is also a role for this enzyme activity in several aspects of cardiovascular disease (30). Although GGT levels may be elevated in patients with NAFLD, there is little data on the frequency and degree of elevation (17).

Noninvasive assessment of fibrosis severity is crucial in the management of patients with NAFLD since its stage is a major determinant of all cause and liver-related mortality (10,31–33). Non-invasive scoring systems based on clinical and biochemical variables are increasingly being used to estimate the degree of hepatic fibrosis without biopsy. In our study, the scores evaluated were effective to exclude advanced fibrosis/cirrhosis, with good Se and NPV, both with the cut-off proposed in our study and with that recommended in the literature. FIB-4 and NFS (AUROC 0.82 and 0.89, respectively) outperformed APRI (AUROC 0.73) for advanced liver fibrosis and cirrhosis with a *P* of 0.04. There was no significant difference between FIB-4 and NFS AUROC’s. The first use of FIB-4 index was in patients co-infected with hepatitis C and human immunodeficiency virus, then the score was validated for NAFLD, with interesting results in studies published from around the word (34). For values higher than 3.25 in a comparison of fibrosis markers in 541 patients with NAFLD, FIB-4 acquired the highest AUROC of 0.80, with NPV and PPV of 90% and 80%, respectively, in predicting advanced fibrosis (35). In our study, FIB-4’s NPV was 87%. Pérez-Gutiérrez and cols., using the same cut-off value for predictions of severe fibrosis, in a Latin population, obtained lower PPV of 26% and 53% Se (36). APRI ratio is an easy and accessible score. Calès and cols. demonstrated an APRI’s AUROC of 0.87 for significant fibrosis in a study of 235 patients with NAFLD (37). NFS is composed of six variables that was formulated using a panel of 733 subjects with NAFLD across diverse international centers. Calès and cols. reported an AUROC of 0.88 for predicting the presence of significant fibrosis (37).

Although the overall accuracy of these scores is moderate, in general, they have high negative predictive values to exclude advanced liver fibrosis, especially in community and primary care settings. Patients with low fibrosis scores are also at a low risk of developing liver-related complications (4). In the medical routine, NFS and FIB-4 are the most commonly used scores (38). Despite displaying good diagnostic efficacy, many patients (30%) fall in-between the lower and upper threshold values (indeterminate results), and many factors such as age, diabetes, and prevalence of fibrosis, among others, may influence their diagnostic performance (10). Sequential combination of these scores with imaging methods such as elastography has been proposed as a diagnostic strategy that could reduce the need for liver biopsies in situations of indeterminate scores results (39).

This study has limitations. First, the design has limitations inherent to cross-sectional studies resulting from atemporal monitoring with high proportion of missing data. Second, our patients had lower rates of advanced fibrosis, and a possible explanation may be the fact that biopsy is avoided in those who have clinical, laboratory and imaging signs compatible with cirrhosis.

There was an association between age, components of the metabolic syndrome such as diabetes, dyslipidemia, hypertension and some biochemical tests (AST/ALT ratio, GGT, platelet count and fasting glucose) and liver fibrosis in patients with NAFLD. As it is a silent disease with substantial heterogeneity of phenotypes, these factors, in combination with non-invasive scores such as FIB-4 and NFS, could help in patient stratification, selection for liver biopsy and identification of who will benefit from early intervention. Besides that, as NAFLD is a highly prevalent disease, with different local realities, studies as this one could be a basis for knowledge of the local reality and provide important information for the development of public health programs.

## References

[1] Chalasani N, Younossi Z, Lavine JE, Charlton M, Cusi K, Rinella M (2018). The diagnosis and management of nonalcoholic fatty liver disease: Practice guidance from the American Association for the Study of Liver Diseases. Hepatology.

[2] European Association for the Study of the Liver (EASL); European Association for the Study of Diabetes (EASD); European Association for the Study of Obesity (EASO) (2016). EASL-EASD-EASO Clinical Practice Guidelines for the management of non-alcoholic fatty liver disease. J Hepatol.

[3] Castera L, Friedrich-Rust M, Loomba R (2019). Noninvasive Assessment of Liver Disease in Patients With Nonalcoholic Fatty Liver Disease. Gastroenterology.

[4] Powell EE, Wong VW, Rinella M (2021). Non-alcoholic fatty liver disease. Lancet.

[5] Eslam M, Sanyal AJ, George J, International Consensus Panel (2020). MAFLD: A Consensus-Driven Proposed Nomenclature for Metabolic Associated Fatty Liver Disease. Gastroenterology.

[6] Younossi Z, Tacke F, Arrese M, Chander Sharma B, Mostafa I, Bugianesi E (2019). Global Perspectives on Nonalcoholic Fatty Liver Disease and Nonalcoholic Steatohepatitis. Hepatology.

[7] Dulai PS, Singh S, Patel J, Soni M, Prokop LJ, Younossi Z (2017). Increased risk of mortality by fibrosis stage in nonalcoholic fatty liver disease: Systematic review and meta-analysis. Hepatology.

[8] Brunt EM, Janney CG, Di Bisceglie AM, Neuschwander-Tetri BA, Bacon BR (1999). Nonalcoholic steatohepatitis: a proposal for grading and staging the histological lesions. Am J Gastroenterol.

[9] Associação Brasileira para o Estudo da Obesidade e da Síndrome Metabólica (Abeso) (2016). Diretrizes Brasileiras de Obesidade.

[10] Vilar-Gomez E, Chalasani N (2018). Non-invasive assessment of non-alcoholic fatty liver disease: Clinical prediction rules and blood-based biomarkers. J Hepatol.

[11] Angulo P, Hui JM, Marchesini G, Bugianesi E, George J, Farrell GC (2007). The NAFLD fibrosis score: a noninvasive system that identifies liver fibrosis in patients with NAFLD. Hepatology.

[12] Sterling RK, Lissen E, Clumeck N, Sola R, Correa MC, Montaner J, APRICOT Clinical Investigators (2006). Development of a simple noninvasive index to predict significant fibrosis in patients with HIV/HCV coinfection. Hepatology.

[13] Lin ZH, Xin YN, Dong QJ, Wang Q, Jiang XJ, Zhan SH (2011). Performance of the aspartate aminotransferase-to-platelet ratio index for the staging of hepatitis C-related fibrosis: an updated meta-analysis. Hepatology.

[14] Krzanowski WJ, Hand DJ (2011). ROC curves for continuous data.

[15] DeLong ER, DeLong DM, Clarke-Pearson DL (1988). Comparing the areas under two or more correlated receiver operating characteristic curves: a nonparametric approach. Biometrics.

[16] Faludi AA, Izar MCO, Saraiva JFK, Chacra APM, Bianco HT, Afiune A (2017). Atualização da Diretriz Brasileira de Dislipidemias e Prevenção da Aterosclerose – 2017. Arq Bras Cardiol.

[17] Sanyal AJ, American Gastroenterological Association (2002). AGA technical review on nonalcoholic fatty liver disease. Gastroenterology.

[18] Salgado W, Santos JS, Sankarankutty AK, Silva Ode C (2006). Nonalcoholic fatty liver disease and obesity. Acta Cir Bras.

[19] Lonardo A, Nascimbeni F, Ballestri S, Fairweather D, Win S, Than TA (2019). Sex Differences in Nonalcoholic Fatty Liver Disease: State of the Art and Identification of Research Gaps. Hepatology.

[20] Klair JS, Yang JD, Abdelmalek MF, Guy CD, Gill RM, Yates K, Nonalcoholic Steatohepatitis Clinical Research Network (2016). A longer duration of estrogen deficiency increases fibrosis risk among postmenopausal women with nonalcoholic fatty liver disease. Hepatology.

[21] Lovejoy JC, Champagne CM, de Jonge L, Xie H, Smith SR (2008). Increased visceral fat and decreased energy expenditure during the menopausal transition. Int J Obes (Lond).

[22] Younossi ZM, Koenig AB, Abdelatif D, Fazel Y, Henry L, Wymer M (2016). Global epidemiology of nonalcoholic fatty liver disease-Meta-analytic assessment of prevalence, incidence, and outcomes. Hepatology.

[23] Chatrath H, Vuppalanchi R, Chalasani N (2012). Dyslipidemia in patients with nonalcoholic fatty liver disease. Semin Liver Dis.

[24] Zhang QQ, Lu LG (2015). Nonalcoholic Fatty Liver Disease: Dyslipidemia, Risk for Cardiovascular Complications, and Treatment Strategy. J Clin Transl Hepatol.

[25] Zhao YC, Zhao GJ, Chen Z, She ZG, Cai J, Li H (2020). Nonalcoholic Fatty Liver Disease: An Emerging Driver of Hypertension. Hypertension.

[26] Toshikuni N, Tsuchishima M, Fukumura A, Arisawa T, Tsutsumi M (2017). Associations of Fatty Liver Disease with Hypertension, Diabetes, and Dyslipidemia: Comparison between Alcoholic and Nonalcoholic Steatohepatitis. Gastroenterol Res Pract.

[27] Khashab MA, Liangpunsakul S, Chalasani N (2008). Nonalcoholic fatty liver disease as a component of the metabolic syndrome. Curr Gastroenterol Rep.

[28] Bedogni G, Miglioli L, Masutti F, Tiribelli C, Marchesini G, Bellentani S (2005). Prevalence of and risk factors for nonalcoholic fatty liver disease: the Dionysos nutrition and liver study. Hepatology.

[29] Angulo P (2007). GI epidemiology: nonalcoholic fatty liver disease. Aliment Pharmacol Ther.

[30] Neuman MG, Malnick S, Chertin L (2020). Gamma glutamyl transferase – an underestimated marker for cardiovascular disease and the metabolic syndrome. J Pharm Sci.

[31] Rafiq N, Bai C, Fang Y, Srishord M, McCullough A, Gramlich T (2009). Long-term follow-up of patients with nonalcoholic fatty liver. Clin Gastroenterol Hepatol.

[32] Hagström H, Nasr P, Ekstedt M, Hammar U, Stål P, Hultcrantz R (2017). Fibrosis stage but not NASH predicts mortality and time to development of severe liver disease in biopsy-proven NAFLD. J Hepatol.

[33] Angulo P, Kleiner DE, Dam-Larsen S, Adams LA, Bjornsson ES, Charatcharoenwitthaya P (2015). Liver Fibrosis, but No Other Histologic Features, Is Associated With Long-term Outcomes of Patients With Nonalcoholic Fatty Liver Disease. Gastroenterology.

[34] Dowman JK, Tomlinson JW, Newsome PN (2011). Systematic review: the diagnosis and staging of non-alcoholic fatty liver disease and non-alcoholic steatohepatitis. Aliment Pharmacol Ther.

[35] Shah AG, Lydecker A, Murray K, Tetri BN, Contos MJ, Sanyal AJ, Nash Clinical Research Network (2009). Comparison of noninvasive markers of fibrosis in patients with nonalcoholic fatty liver disease. Clin Gastroenterol Hepatol.

[36] Pérez-Gutiérrez OZ, Hernández-Rocha C, Candia-Balboa RA, Arrese MA, Benítez C, Brizuela-Alcántara DC (2013). Validation study of systems for noninvasive diagnosis of fibrosis in nonalcoholic fatty liver disease in Latin population. Ann Hepatol.

[37] Calès P, Lainé F, Boursier J, Deugnier Y, Moal V, Oberti F (2009). Comparison of blood tests for liver fibrosis specific or not to NAFLD. J Hepatol.

[38] Sheka AC, Adeyi O, Thompson J, Hameed B, Crawford PA, Ikramuddin S (2020). Nonalcoholic Steatohepatitis: A Review. JAMA.

[39] Mózes FE, Lee JA, Selvaraj EA, Jayaswal ANA, Trauner M, Boursier J (2022). Diagnostic accuracy of non-invasive tests for advanced fibrosis in patients with NAFLD: an individual patient data meta-analysis. Gut.

